# Does family confidence bridge child functioning and caregiver burden in early intervention?

**DOI:** 10.1371/journal.pone.0321997

**Published:** 2025-05-12

**Authors:** Gabriel Martínez-Rico, Pau García-Grau, Margarita Cañadas-Pérez, Rómulo J. González-García

**Affiliations:** Campus Capacitas, Catholic University of Valencia “San Vicente Mártir”, Valencia, Spain; Qatar University College of Nursing, QATAR

## Abstract

*Background*: Enhancing caregivers’ confidence and competence in early childhood intervention is a priority, focusing on collaboration between professionals and families to improve family well-being and quality of life. Caregiver burden is crucial in early childhood intervention support services aimed at promoting family well-being and functioning. However, contextual factors and child-related variables may affect caregiver burden. We examined the relationships between family confidence, caregiver burden, and child functioning. *Method*: A total of 169 Spanish families receiving early intervention services participated in a cross-sectional survey study. Data were analyzed applying single and multiple mediation analyses to examine the influence of socio-demographic variables on family confidence and caregiver burden, as well as the mediating role of family confidence between child functioning and caregiver burden. Results: The study found that child functioning does not directly impact caregiver burden but significantly influences it through family confidence. Confidence in helping the child was a relevant mediator of the impact of child functioning on caregiver burden. However, confidence in helping the family was a mediator across all dimensions of caregiver burden. *Conclusions*: Higher family confidence predicts lower caregiver burden in early childhood intervention. Confidence in helping family functioning is key to mitigating the negative impact of child functioning on caregiver burden. Practical implications of these findings suggest that early childhood intervention services should focus on capacity-building through collaborative and family-centered practices, empowering families to enhance their confidence, and reduce caregiver burden.

## 1. Introduction

The quality of early childhood intervention (ECI) services is a key determinant of both family well-being and the child’s development and functioning in the first years of life. The main objective of service delivery is to optimize various family outcomes, such as increasing family confidence and competence (two dimensions of *family capacity* according to [1], increasing child functioning and social participation, increasing family autonomy, as well as reducing parental stress and caregiver burden. Globally, all these outcomes, especially improving family confidence and competence are directly linked to family quality of life, which should be used as quality indicator of ECI services [2–5].

Several studies have analyzed the variables that impact caregiver burden and quality of life [6–8]. These studies have analyzed caregiver burden in the field of elderly care [9], health and mental health [10,11], oncology patient care [12] or the study of parental stress in the care of children with support needs [13]. Despite these contributions, research has not advanced sufficiently in the field of ECI, being necessary to deepen the analysis of the variables that explain caregiver burden and other aspects of caregiver well-being, as well as their interaction with other variables such as: child functioning, family confidence, support network, empowerment, and family quality of life.

In this article, we discuss the complex relationships that combine in ECI services: interventions aimed at decreasing caregiver burden, increasing family confidence, and considering influential factors related to child and family functioning.

### 1.1. Caregiver burden in ECI

In a broad sense, caregiver burden has been used in the field as a construct that groups together the physical, emotional, social, or financial problems experienced by family members in the caregiving situation. In a more operational sense, this construct is defined, from a multidimensional approach, around self-efficacy expectations, the impact of caregiving and interpersonal relationships [8,14,15] and is the result of the caregiver’s perception and cognitive appraisal to adapt to the stressors derived from the parenting situation of a child with special needs [6].

In the early years of life of children with support needs (i.e., children with a disability, developmental delay or at-risk situation who experience needs of support), families face numerous unforeseen situations characterized by uncertainty, anxiety, parental stress or caregiving demands [16]. Families must select ECI services that meet their needs, determine the supports their children require, and identify the most suitable schooling. Additionally, it is essential to assess the nature of support needs and any potential developmental concerns. Collectively, these factors influence the perception and cognitive assessment of caregiver burden [4]. This fact is highly relevant given that research has shown a very close relationship between caregiver burden and FQoL [17–19]. Different research has studied this relationship showing that perceived caregiver burden negatively predicts FQoL [7,20]. In parallel, other research has reflected a negative relationship between family burden and “life satisfaction” of parents who have children with disabilities [19]. These results indicate that family confidence and caregiver burden are two variables that significantly affect FQoL. ECI services should strengthen their programs to increase family confidence and reduce the impact of caregiver burden [7].

In this context, a greater sense of parental self-efficacy is related to a lower level of stress caused by the burden [21]. Parental self-efficacy was found to increase as the collaboration between the professional and the family strengthens, especially showing greater improvement when the collaboration is closer and when caregivers are more involved in the intervention. In addition, ECI practices, have been shown to have a positive impact on the perception of caregiver burden in early childhood [22,23]. Among other service features, home visits by professionals have evidenced a decrease in caregiver burden resulting from family visits to centers [24].

### 1.2. Family confidence and competence in ECI

Confidence and competence are both dimensions of family capacity [25]. Family confidence, a result of support-based services, is a key aspect of self-efficacy, reflecting caregivers’ belief in their ability to promote their child’s development. Through capacity-building approaches in ECI, parental competencies are developed, and family confidence in these skills predicts how effectively they implement interventions [26]. This leads to better child and family outcomes such as child functioning and development and reduced parenting stress [2,15].

In the context of our study with families in ECI, family confidence can be seen as the sense of mastery and efficacy in carrying out daily parenting tasks [27,28]. Other authors state that it refers to both the primary caregiver’s perceived confidence in helping the family with routines and dynamics and the perceived confidence in helping the child with functioning in routines and social participation spaces [7,20,29–33]. From this approach, family confidence is a construct that is directly associated with a significant improvement in interactions between primary caregivers and children with special needs [2,5].

In this context, parenting competences, and the family’s confidence in those competences, are directly related to a higher family quality of life [5]. These authors found that certain characteristics of ECI services, such as multiple professionals involved in the family life, are related to lower family confidence and a worse perception of the child’s functioning [5]. In this sense, a transdisciplinary approach, through a primary service provider connecting the family with other professionals can contribute to reducing the impact of stressors and perceived caregiver burden [34]. Thus, promoting the confidence and competence of primary caregivers constitutes an indicator of achievement and success of family-centered support programs [34,35] especially with the presence of a primary service provider [36–38].

Other studies have demonstrated that lower family confidence and competence among parents are associated with greater difficulties in managing problematic behaviors in their children [1,39]. In addition, greater parental confidence contributes to generate more opportunities for social participation and greater management of daily routines, which allows expecting a lower caregiver burden and greater overall well-being [26]. Family confidence was found to mediate the negative effect that caregiver burden or the child’s level of support needs had on FQoL [7]. This effect also extends to other sociodemographic variables such as family income or caregiver gender, modulating their impact on family well-being and quality of life in early childhood [7].

Ultimately, it should be noted that, among other objectives, ECI services aim to increase family confidence by providing the necessary supports at this early stage of the child’s life [4]. Family confidence and competence can therefore be understood as an expected outcome of ECI services [1,19].

### 1.3. Child functioning and sociodemographic variables

In addition to family-level variables, there are child-related factors that can impact caregiver burden. Several studies found that child variables such as the child’s degree of social participation, functioning in routines, age or supports needs have a direct impact on caregiver burden and family quality of life [7,40,41]. Likewise, support needs, behavioral problems, and the need for intensified supports impact the primary caregiver’s perception of caregiving overload [35,42,43].

The relationship between child functioning, family confidence, and caregiver burden has also been partially investigated. For example, different authors have established an inverse relationship between child functioning -severity of the disability or amount of need for supports- and family well-being and quality of life [18,42,44]. Other studies have concluded that child support needs (level of child functioning) do not predict family quality of life by themselves but are positively related to caregiver burden [7].

On the other hand, research also shows that if ECI services contribute to increased family competence and confidence, coupled with greater perceived parental support, there is a significant improvement in child functioning [45,46]. This strong association between the child’s level of functioning and lower perceived caregiver burden has been widely described [47].

Likewise, family-level sociodemographic variables such as being employed [18,42], a greater family income [43,48,49], the presence of two adults living in the household [17,50], greater caregiver education level [35] or more family-centered supports provided by ECI services [40] have been shown to be variables that significantly impact positive child functioning, and greater FQoL.

In contrast, enhanced child functioning and age have been positively correlated with more elevated levels of caregiver burden, which signifies a more dynamic familial environment and the responsibilities that must be undertaken [51], as this often necessitates increased care and attention as children develop and interact [52].

Conversely, time spent by fathers with the child has been associated with a reduction in the perceived parenting burden within the family, concurrently resulting in an enhancement of the child’s functioning perception [51].

Building on these premises, this study positions family confidence as a cornerstone in ECI. A confident family is better equipped to support their child’s development in daily life. Family confidence, as a predictor of FQoL, has the potential to mitigate the adverse effects of caregiver burden. Additionally, while a child’s level of functioning is known to enhance FQoL directly, the demands of caregiving can impose significant strain on families. By examining the interplay between these variables, particularly how family confidence can mediate the impact of caregiver burden on FQoL, this research aims to identify pathways to support families more effectively in ECI contexts [2,4,7].

The focus of the present study challenges the traditional assumptions in ECI research by demonstrating that child functioning does not directly predict caregiver burden but instead operates through its impact on parental confidence. This highlights the need for implementing a capacity-building approach within ECI that emphasize the empowerment of parents.

Within the framework described above, the study presented here has two research objectives:

- To analyze the relationship between family confidence and child functioning as predictors of caregiver burden.

- To analyze the influence of the child’s level of functioning on caregiver burden with and without the mediating influence of family confidence.

## 2. Materials and methods

### 2.1. Participants

A total of 169 Spanish families with children from birth to age six experiencing disability, developmental delay, or at risk of disability or biopsychosocial vulnerability participated in Early Childhood Intervention (ECI) services across five Autonomous Communities (Regions). The majority of families lived in Valencian Community (85.6%), followed by Canary Islands (6.6%), Andalusia (3.3%), and La Rioja (2.6%).

Regarding the family-level variables, the majority of respondents were mothers, (73.97%), followed by fathers (15.75%). Grandparents represented the smallest group at 1.37%. Regarding employment status, the largest portion of respondents are employed full-time (47.54%), with part-time employees comprising 24.59%, and the unemployed -but looking for a job- representing the smallest group at 9.84%. An 18.03% of respondents were homemakers or pension recipients. The monthly salary of each family unit was less than €1800 in 21.19% of cases, while in 78.81% of cases it was higher than €1800 per month.

Among child-level variables (age M = 36.72 months, SD = 7.51), 63.93% of the children did not have a diagnosed disability at the moment, rather, they had developmental delays or were at-risk due to bio-psycho-social factors. A total of 17.21% were in the process of being diagnosed, and 16.39% had a low level of disability. Only 1.64% had severe level of disability followed by 0.82% with moderate level of disability. There was only one missing case (0.82%) that did not report the disability condition. Over 78% percent of the families reporting marital status were married or living with a partner, whereas the 18.48% were single and 3.36% were divorced.

Regarding the service-level variables, the most frequent weekly sessions was once a week (70.25%), followed by every other week 15.70% and only 4.13% of families received more than two sessions per week.

Participants were contacted via email through their early childhood intervention programs with a link to the anonymous on-line survey. It took about 20 minutes to complete and the survey included the questionnaires listed in the instruments section. A written informed consent was embedded at the beginning of the survey and was a required field to access the questionnaires. Only the researchers could access, download, code and analyze the data. Any family receiving services from the participating ECI program was eligible. The link was available from October 1 to December 31, 2023. The study was conducted in accordance with the Declaration of Helsinki an Institutional Review Board approval was obtained from the Ethics Committee of the Catholic University of Valencia (protocol code CEI/UCV/2018–2019/111).

### 2.2. Instruments

#### 2.2.1. Child functioning in everyday routines.

To obtain a family’s measure of child performance in routines we used the “child functioning” subscale of the family quality of life -FEIQoL- scale [30]. It takes five minutes to complete and includes 13 items on a five-point Likert scale. Families rate the child’s performance in everyday situations from 1 = poor to 5 = excellent. This subscale has been validated through several confirmatory factor analyses and subsequently through Rasch analysis [50,53] in a Spanish sample within the ECI population (e.g., families with children with a disability, delay, or bio-psycho-social risk). This instrument was chosen for its valuable family input on child functioning in ECI and its cultural and linguistic appropriateness. Within the Spanish specific context it showed high internal consistency (α = .89) in [50] and KR-20 = .99 in [53]. In our sample, internal consistency values were α = .934 and ω = .934.

#### 2.2.2. Caregiver burden.

Zarit’s Caregiver Burden Interview (CBI) [15] adapted to Spanish by [8] and reduced by [54], was used for this study. This adapted version of the scale is composed of 12 items on a 5-point Likert scale (1 = Never to 5 = Always). This tool has been widely used and numerous adaptations and proposals for reduced versions have emerged in a multitude of fields because it can be easily adapted for different populations. We used the reduced 12-item version because of its length and high internal consistency of the scores in Spanish (α = .92), in addition to the applicability of the items to families in ECI services in Spain. The internal consistency of the scores in our sample was α = 0.87 for the global caregiver burden score.

#### 2.2.3. Family confidence in early childhood intervention.

The Family Confidence in Helping with Child Functioning in Routines and Family Functioning (Fam-Con; [30]. It is a two-dimension tool:

(a)  Confidence in helping the child in everyday routines (CHC): Families rate the degree of confidence in helping the child participate, be independent, communicate, and behave appropriately in a set of 5 daily routines (mealtimes, play times, outings, bath times, and going-to-bed time). It consists of 20 items on a Likert scale of 1 = I’m not quite sure how I can help with this to 4 = I have complete confidence in how to help my family with this.
(b)  Confidence in helping with family functioning (CHF). This section has 18 items measuring family confidence in helping themselves and the rest of the family with aspects related to family functioning. It includes indicators related to informational, emotional, and material support, as well as indicators to rate caregiver’s confidence in help with family needs. This part of the Fam-Con uses the same Likert scale.

In previous studies in Spain [5], the internal consistency of the scores was Cronbach’s Alpha = 0.96 for CHC and 0.94 for CHF.

In our study, similarly high values of internal consistency scores were found for CHC (α = 0.95) and for CHF (α = 0.93). No other tool, to our knowledge examines both child and family dimensions while being structured around family routines, which led the researchers select this tool. In addition, it showed excellent internal consistency of the scores in both dimensions.

### 2.3. Data analysis

Descriptive analyses were conducted to characterize the participants and to evaluate the scoring patterns of the measures used. This included calculating means, standard deviations, and the range of scores (minimum and maximum) at both the overall and dimension levels. For this purpose, we used SPSS v.25 (IBM, 2019). To calculate the internal consistency of the scores, alongside Cronbach’s alpha, McDonald’s omega (ω) was also used due to the ordinal nature of the Likert-type scores.

Furthermore, ANOVA and t tests were conducted to describe the scores for family confidence, child functioning, and caregiver burden based on sociodemographic variables of interest such as income and employment. A 95% confidence interval was used, with statistical significance accepted at p < .05. Effect sizes were calculated using Cohen’s d, with values of.20,.50, and.80 indicating small, medium, and large effect sizes, respectively [55]. Analyses of the relationships between continuous variables in the study were conducted using Pearson correlations. Finally, in order to address our second objective, a mediation analysis was carried out in which child functioning was the predictor, family confidence the mediator variable, and caregiver burden was the outcome variable. Specifically, we analyzed the direct effect of child functioning on caregiver burden, as well as the indirect effect through family confidence. We also calculated the total effect considering the impact of the predictor on the dependent variable without removing the effect of the moderator in the equation. We used a robust mediation method in JASP v.0.16.4 JASP Team, 2024, following the procedures of similar work on the topic [7]. Finally, a multiple mediation analysis (two mediators) was conducted for a deeper detail and understanding of how each of the three caregiver burden factors -dependent variables- (Impact of Care, Interpersonal Relationships and Self-efficacy) are predicted by child functioning -predictor- through the two dimensions of family confidence -mediators- (Confidence in Helping the Child -CHC-, and Confidence in Helping the Family -CHF-). In other words, we tested how child functioning predicts all three domains of caregiver burden individually and through each of the dimensions of family confidence.

## 3. Results

The scores of caregivers in all constructs of study were analyzed through mean scores and standard deviations. With regard to family confidence scores it stands out that both confidence in helping the family (M = 2.89) and confidence in helping the child (M = 2.91) were around three points out of four (SD = .59 and 0.66 respectively), indicating that families had moderate confidence. Regarding the child’s functioning, an average score of 3.24 (SD = 0.81) was observed. This result indicated that the children’s average functioning was adequate, with a neutral score among the 5 response categories.

Regarding the caregiver burden dimensions, the Self-efficacy dimension was the most highly scored, with average values of 2.75 (SD = 1.14). The lowest score in the caregiver burden dimensions was in Interpersonal Relationships, with an average score of 1.69 (SD = 0.66). Impact of care showed a mean score of 2.45 (SD = 0.94).

The overall caregiver burden score was M = 2.19 (SD = 0.73). In addition to the average score, for greater comparability of the results regarding the overall burden score, the total score was also computed through the sum of the 12 items (following [8]). The results indicated a total score of 26.32 (SD = 8.74) (Table 1).

**Table 1:** Means, standard deviations, minimum and maximum scores on family confidence, child functioning, and caregiver burden.

	N	M	SD	Min	Max
**Family Confidence**					
Confidence in Helping the Family (CHF)	143	2.892	0.590	1.167	4.000
Confidence in Helping the Child (CHC)	143	2.911	0.663	1.000	4.000
**Caregiver Burden**					
Impact of Care	141	2.453	0.942	1.000	5.000
Interpersonal Relationships	141	1.687	0.660	1.000	3.600
Self-efficacy	141	2.745	1.138	1.000	5.000
Overall caregiver burden	135	26.319	8.737	12.000	49.000
**Child Functioning**	141	3.242	0.812	1.467	5.000

To describe these scores in greater detail according to the sociodemographic variables, analyses of variance were carried out. The results of the independent samples t test comparing family monthly income above and below 1,800 euros showed that there were statistically significant differences with medium effect sizes in both CHC [t(109) = 2.27, p = .025, d = .523] and CHF [t(109) = 2.82, p = .006, d = .651] dimensions in favor of the group of families with incomes above 1800 euros. There were no relevant differences in the perceptions of caregiver burden according to the family income (p < .05).

Regarding differences in employment, the results of the ANOVA test indicated that there were no differences in the scores of child functioning [F(3, 101) = 2.093; p < .05, η² = .059], caregiver burden [F(3, 109) = 0.643; p < .05, η² = .017], family confidence in helping the child [F(3, 101) = 2,093; p < .05, η² = .059] or confidence in helping the family [F(3, 111) = 1.254; p < .05, η² = .033].

Subsequently, the correlations between family confidence, caregiver burden, and child functioning were analyzed with the following sociodemographic variables: age, number of adults and children living in the home, and number of weekly intervention sessions (Table 2).

**Table 2:** Correlations between caregiver burden, family confidence, and child functioning with quantitative sociodemographic variables.

Variable	1	2	3	4	5	6	7	8	9	10	11
1. CHF	**—**										
2. CHC	0.583***	—									
3. Burden	-0.480***	-0.136	—								
4. Impact of care	-0.383***	-0.042	0.931***	—							
5. Interp. Rel.	-0.427***	-0.159	0.880***	0.749***	—						
6. Self Effi.	-0.307***	-0.098	0.592***	0.369***	0.331***	—					
7. Child Funct.	0.102	-0.188*	-0.083	0.086	-0.050	-0.062	—				
8. Age	0.059	0.143	0.092	0.102	0.086	0.020	0.104	—			
9. #Adults	-0.068	-0.104	-0.089	-0.188	-0.050	0.137	0.032	-0.058	—		
10. # Children	0.021	-0.117	-0.055	-0.083	-0.062	0.037	0.054	0.072	0.041	—	
11. Sessions/Week	0.063	-0.131	-0.001	0.039	0.065	-0.189	-0.014	0.027	-0.023	-0.006	

* p < .05, ** p < .01, *** p < .001.

The results of these correlations indicated that a greater number of weekly sessions was negatively related to the self-efficacy dimension of caregiver burden. This relationship was statistically significant and indicated that a greater number of sessions was associated with lower self-efficacy perceptions (r = -.19; p < .05). Likewise, a greater number of adults in the household was related to lower perceptions of impact on care, a result indicated by a negative correlation between both variables (r = -.19; p < .05). The number of children living in the place and the age of the child were not related to family confidence, caregiver burden, or child functioning.

With regard to the scores on the constructs of the study and their linear relationships, it should be noted, above all, that family Confidence in helping the family (CHF) is statistically significant and negatively related to the caregiver’s burden across its three dimensions, as well as to the total burden (p < .001 in all cases). The result indicated that lower levels of confidence in helping the family are associated with greater perceptions of caregiver burden in all its dimensions. On the other hand, it is noteworthy that the correlations between the family confidence with helping the child (CHC) and caregiver burden dimensions were not statistically significant (p > .05).

Finally, a statistically significant negative relationship between the child’s functioning and confidence in helping the child (CHC) was found, indicating that caregivers with children with higher functioning also perceive lower confidence in managing this functioning (r = -.19; p < .05).

After analyzing these linear relationship patterns and to respond to our third objective, the mediating role of confidence in helping the family (CHF) in the relationship between the child’s functioning and the caregiver’s burden was analyzed (Table 3). The results indicated that child functioning, by itself, does not have a relevant effect on caregiver burden, indicated by a non-statistically significant direct effect (b = -1.18; z = -1.40; p < .160).

**Table 3:** Mediation of family confidence in the relation between child functioning and caregiver burden.

									95%CI	
**IV**		**Mediator**		**DV**	**Estimate**	**SE**	**z**	**p**	**Lower**	**Upper**
**Direct effect**										
Child Funct.		→		Burden	-1.178	0.839	-1.404	0.160	-2.824	0.467
**Indirect effect**										
Child Funct.	→	CHF	→	Burden	-1.769	0.532	-3.327	< .001	-2.812	-0.727
**Total effect**										
Child Funct.		→		Burden	-2.948	0.890	-3.312	< .001	-4.692	-1.203
**Path coefficients**										
CHF		→		Burden	-7.134	1.262	-5.652	< .001	-9.608	-4.660
Child Funct.		→		CHF	0.248	0.060	4.106	< .001	0.130	0.366

* p < .05, ** p < .01, *** p < .001.

However, the analysis of indirect effect, through the confidence in helping the family, is highly significant (b = -1.77; z = -3.33; p < .001). The relationship in this case turns out to be negative, indicating that higher levels of child functioning and confidence in helping the family predicted lower total caregiver burden scores.

Considering the effect of the influence of family confidence, the total effect of child functioning on caregiver burden turns out to be statistically significant (b = -2.95; z = -3.31; p < .001). This result indicated that lower levels of child functioning predicted greater caregiver burden when confidence in helping the family is in the equation.

The absence of a direct effect of child functioning on caregiver burden and finding a statistically significant impact through confidence in helping the family (CHF) indicates that the mediating role of confidence turns out to be a complete mediation.

**Fig 1:**
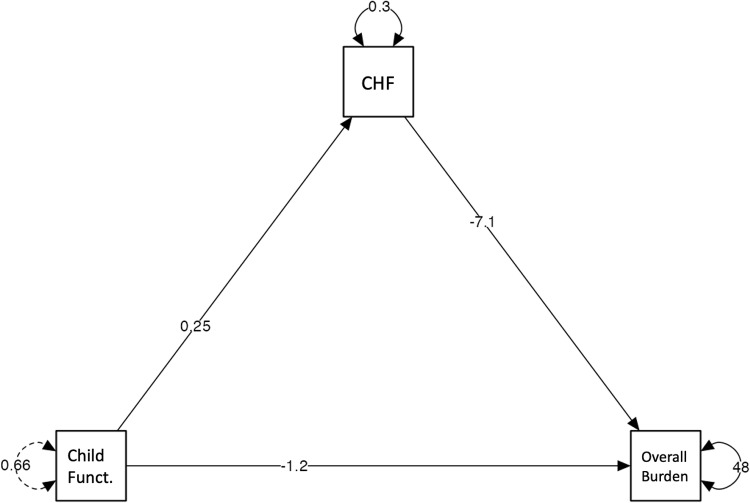
Relationship between the above-mentioned variables.

Regarding the individual regressions of the model, the influence of confidence in helping the family, by itself, predicted a lower burden on the caregiver, resulting in the most significant regression of the model (b = -7.13; z = -5.65; p < .001). In addition, a child’s greater functioning, by itself, predicted greater perceptions of confidence in helping the family (b = 0.25; z = 4.11; p < .001). Fig 1 shows the relationship between the above-mentioned variables.

As the next level of inquiry and to obtain more detail on the prediction of caregiver burden domains through family confidence, multiple mediation across the two dimensions of family confidence was conducted (Table 4).

**Table 4:** *Multiple mediation of family confidence dimensions in the relation between child functioning and caregiver* burden dimensions.

									95%CI	
					**Estimate**	**SE**	**z**	**p**	**Lower**	**Upper**
**Direct effects**										
Child Funct.		→		Imp. Of Care	-0.175	0.095	-1.854	0.064	-0.361	0.010
Child Funct.		→		Interpersonal Rel.	-0.158	0.066	-2.389	**0.017**	-0.288	-0.028
Child Funct.		→		Self-Efficacy	0.014	0.123	0.110	0.912	-0.228	0.256
**Indirect effects**										
Child Funct.	→	CHF	→	Imp. Of Care	-0.223	0.068	-3.267	**0.001**	-0.357	-0.089
Child Funct.	→	CHC	→	Imp. Of Care	0.114	0.050	2.288	**0.022**	0.016	0.212
Child Funct.	→	CHF	→	Interpersonal Rel.	-0.129	0.043	-3.017	**0.003**	-0.213	-0.045
Child Funct.	→	CHC	→	Interpersonal Rel.	0.040	0.031	1.313	0.189	-0.020	0.101
Child Funct.	→	CHF	→	Self-Efficacy	-0.193	0.072	-2.693	**0.007**	-0.333	-0.052
Child Funct.	→	CHC	→	Self-Efficacy	0.102	0.060	1.713	0.087	-0.015	0.219
**Total effects**										
Child Funct.		→		Imp. Of Care	-0.284	0.098	-2.904	**0.004**	-0.476	-0.092
Child Funct.		→		Interpersonal Rel.	-0.247	0.067	-3.692	**< .001**	-0.378	-0.116
Child Funct.		→		Self-Efficacy	-0.077	0.120	-0.647	0.518	-0.312	0.157
**Total indirect effects**										
Child Funct.		→		Imp. Of Care	-0.109	0.058	-1.864	0.062	-0.223	0.006
Child Funct.		→		Interpersonal Rel.	-0.088	0.038	-2.335	**0.020**	-0.163	-0.014
Child Funct.		→		Self-Efficacy	-0.091	0.063	-1.440	0.150	-0.215	0.033

Regarding direct effects, it stands out that child functioning only had a relevant negative effect on interpersonal relationships (b = -.16. z = -2.39, p = .02). There was no statistically significant direct effect between child functioning and Impact of care or Self-efficacy.

Indirect effects through family confidence in helping with family functioning (CHF) were relevant for the prediction of the three dimensions of caregiver burden. All the effects were inverse, indicating that higher child functioning predicts higher CHF and therefore a lower score on impact of care (b = -.22. z = -3.7, p = .001), interpersonal relationships (b = -.13. z = -3.02, p < .01), and self-efficacy (b = -.19. z = -2.69, p < .01).

Indirect effects through the confidence in helping the child (CHC) were not relevant to predict the dimensions of interpersonal relationships or self-efficacy (p < .05 in both cases). However, there was a statistically significant indirect effect to predict the impact of care (b = -.11. z = 2.29, p < .05). In this case, the positive sign of the beta indicated that a higher functioning of the child predicted a higher score in the dimension of impact on care through the CHC mediator (Table 4).

The total effects of child functioning on caregiver burden dimensions were statistically significant for predicting the impact of care (b = -.28. z = -2.90, p < .01) and the interpersonal relationships (b = -.25. z = -3.69, p < .001). In terms of predicting the self-efficacy dimension, however, the effects were not relevant. The significant and inverse results indicate that a greater functioning of the child, considering family confidence (but not through it) and predicts lower perceptions of caregiver burden in these two dimensions.

**Fig 2:**
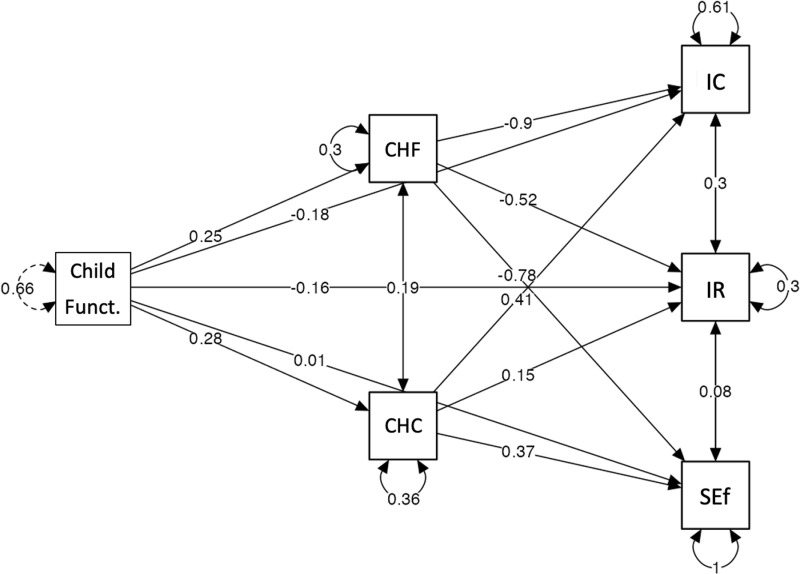
Summary of path coefficients of the multiple mediation model.

Finally, the joint effect of both mediators was analyzed through the total indirect effects, finding that, together, they only predicted the interpersonal relationships inversely (b = -.09. z = 2.34, p < .05). This result indicated that the greater the child’s functioning, the greater the family confidence in both dimensions, and this predicted a lower caregiver burden score relative to interpersonal relationships. Fig 2 illustrates the summary of path coefficients of the multiple mediation model.

## 4. Discussion

The present study analyzed child functioning, family confidence and caregiver burden in families in ECI services in Spain. In addition, we analyzed the dynamics between these variables and tested the mediating role family confidence. Specifically, the novel aspect of the study is that we analyzed the influence of child functioning on caregiver burden through family confidence and found that there is no direct effect, but rather we found an indirect effect through family confidence.

Overall, the scores on caregiver burden showed average levels. These results are consistent with previous research in Spain measuring caregiver burden in families in early childhood intervention [7] and with the original study validating the 12-item version of the scale [8]. With regard to family confidence, we found moderate confidence in both dimensions of the scale, similar to those obtained by [5]. In addition, child functioning scores were also similar to recent findings by [53] also with Spanish families.

While employment status did not seem to impact caregiver burden or family confidence, higher family income levels were associated with lower levels of burden among parents of children in ECI programs. This suggests that financial stability may play a crucial role in alleviating caregiver stress. Previous research has shown that socioeconomic status could be a critical determinant of parental well-being and stress levels [56,57]. Also, [58] stated that stability can provide families with greater access to resources, services, and support systems, which can mitigate the challenges associated with caregiving. Therefore, it is important for ECI programs pay attention to families’ economic resources when designing support interventions. Ensuring that families have access to adequate financial resources as well as connections with other social community resources could potentially reduce the overall burden and improve outcomes for both children and caregivers.

We also found that the greater the number of adults in the household, the lower the impact of care. More adults in the home can help with the distribution of caregiving responsibilities thus alleviating the demands placed on a single caregiver, thereby reducing stress and burnout [59]. This is also related with other studies finding single-parent families perceiving their overall family quality of life lower as compared with families with more adult family members [18].

We also found greater number of weekly sessions correlated with lower self-efficacy scores. These results align with previous studies finding multiple sessions with multiple professionals are associated with higher family confusion and stress due to crossed messages and feelings of lack of coordination [60], and with lower perception of helpfulness of ECI services [61]. The fact that no statistically significant relationships have been found between child functioning and age may be indicative that, in this early intervention population, delays in child functioning are more apparent with age, and conversely, age is not related in this case to higher levels of functioning. This finding can have a direct implication for practice related to individualized intervention planning. If age is not a strong determinant of functioning levels, practitioners may need to focus more on individualized assessments and interventions rather than assuming linear progressions based on age.

Our findings indicate that child functioning alone does not directly predict caregiver burden. Instead, it is the reduction in family confidence, resulting from poor child functioning, that can lead to an increased burden on caregivers. This result does not align previous research stating that a low -or problematic- child functioning predicts a greater stress for caregivers [62]. This inconsistency might be explained by our mediation model’s control of the effect of family confidence when calculating the direct effect of child functioning on caregiver burden. To our knowledge, no other studies in ECI have isolated such effect controlling for family confidence. Further studies are needed to understand the role of family confidence in the effects of child variables on caregiver burden perceptions. In general, disability-related studies found that lower child functioning is associated with greater caregiver burden [63] and lower well-being and family quality of life [42]. In addition, families of children who have high demands or needs for support can have consequences in the day-to-day life that prevent them from enjoying a social life of relief and stress release that has a positive impact on dimensions of caregiver burden such as Interpersonal Relationships [64].

This highlights the crucial role of family confidence in the caregiving dynamic. Previous studies highlight the importance of family confidence and self-efficacy in managing the demands of caregiving for children with support needs [62,65].

Although the impact of caregivers’ self-efficacy beliefs on caregiver burden has been extensively studied in health and mental health contexts [10,12,66,67], this relationship has received comparatively less attention in the field of early childhood intervention. association between greater parenting self-efficacy and lower levels of parental anxiety and depression [68,69], positive child outcomes [39,70], and increased use of promotive and responsive parenting strategies, particularly in the context of environmental and social challenge [71,72].

Confidence in helping the family was the most important mediator above confidence in helping the child in our study. Other studies such as [7] or [5] also found that the family dimension of Fam-Con had a greater influence on FQoL, an outcome that is expected to be inverse of parenting burden and is a direct consequence of an improvement in parental confidence and competence experienced after receiving support services [2].

Confidence in helping the family is a relevant mediator for all three dimensions of caregiver burden, whereas confidence in helping the child is only relevant for predicting the interpersonal relationship dimension. This result suggests that the impact of caring for a low-functioning child on self-efficacy and caregiving is not as relevant as that on caregivers’ interpersonal relationships. This may be indicative of interpersonal relationships being more dependent on confidence in helping the child than other dimensions of caregiver burden. However, more studies are needed with this factor structure of caregiver burden proposed by [8] to further understand the variables that may affect the different components of burden.

In accordance with Bronfenbrenner’s ecological systems theory, the mediating role of family confidence in the relationship between child functioning and caregiver burden illustrate the dynamic interaction among elements of the microsystem and mesosystem. By enhancing parental confidence through family-centered interventions in ECI, caregivers may perceive themselves as more adept at incorporating strategies into their daily routines, which, in turn, could alleviate the influence of the child’s level of functioning on caregiver burden. Policies and practices that emphasize family-centered approaches in ECI, such as collaborative approach, capacity-building interactions or proactive parental participation, could further amplify these effects by nurturing resilience and diminishing stressors at both the mesosystem and exosystem levels.

### 4.1. Limitations

The present study should acknowledge some limitations. First, the sample size could be considered a limitation due to the unequal representation of participants in different regions. Some regions, such as the Valencian Community, had greater participation. Thus, this could affect to the generalization of results. However, the procedures and statistical techniques that were used —robust and appropriate for these cases— and the pattern of results were similar to those found in the literature on caregiver burden and family confidence (i.e., [7]), making this study an interesting contribution. The results should be taken as a promising first step in analyzing the mediating role of family confidence between child-related variables and caregiver burden. We encourage researchers to replicate this study in different contexts with a larger sample size. Furthermore, the analysis did not account for cultural disparities and variations in regional policies. This may be regarded as a limitation and could influence practical implications, particularly in terms of building family confidence implementing family-centered practices. While other studies have analyzed burden as the predictor of variables such as quality of life, the present study has put the attention in caregiver burden as the outcome, and we analyzed the role of family confidence as a buffer variable against this negative effect of the impact of a low child functioning.

### 4.2. Implications

The most significant implication is the need for services to measure and monitor family confidence and caregiver burden. Regularly monitoring these constructs can help programs identify potential areas of need, thereby serving not only as a final evaluation metric but also as a tool for planning and reviewing processes.

The consequences on support services of our results have a clear direction towards the implementation of collaborative practices and family empowerment. Reducing family burden is a relevant outcome of ECI settings. Research indicates that specific activities such as mindfulness can be beneficial, and services could encourage family participation in these activities. Such activities have proven effective in reducing stress among parents of children with disabilities [73,74].

However, because or results identified family confidence as a complete mediator between child functioning and caregiver burden we can recommend services to implement a collaborative capacity-building approach, with special attention to building family confidence. The influence of family confidence in helping the family functioning was even more relevant to the confidence in helping the child. This has a clear implication towards paying special attention to addressing family needs and priorities. This could be implemented in different stages of the intervention process (considering family priorities during needs assessment, evaluation processes, functional planning and also implementation of interventions). As [75] found, addressing more family-level goals (whether related to the child or not), have been related to better child performance.

Additional results from our study, such as the association between more weekly sessions and lower self-efficacy, and the finding that families with lower income feel less confident, suggest that services could, first, avoid overloading families with multiple weekly sessions. In addition, implementing a primary service provider (PSP) could help with both reduce the number weekly sessions and improve coordination to reduce parental stress [76] and improve child functioning and family quality of life [34]. A PSP is the main professional who provides support to the family and is the link between the family and the rest of ECI team specialists if needed, according to the needs. Second, services could also identify families who are more vulnerable and build their confidence not only with helping their child but also in enhancing overall family functioning. Given that insufficient economic resources can exacerbate caregiver burden, it is crucial for services to ensure strong connections with community resources tailored to each family’s needs. Furthermore, targeted interventions should be developed to support these families, addressing both their immediate and long-term challenges. By fostering a holistic approach, services can help mitigate the stressors associated with economic hardship and enhance the overall well-being of the family unit. Ultimately, implementing a family-centered approach (i.e., focusing on families’ identified needs and priorities, building their capacity, establishing positive and collaborative family-professional partnerships, designing functional participation-based intervention plans, and supporting families in natural environments rather than in multiple visits to the clinic), could help services have an important impact in both reducing caregiver burden and increasing family confidence and overall wellbeing.

#### Conclusions.

It can be concluded in this study that the child’s functioning alone does not negatively predict caregiver burden in early intervention, contrary to previous assumptions. Rather, it is the low levels of family confidence - that ultimately predict greater caregiver burden in early intervention. By isolating the effect of this variable using two different mediation analysis, the present study confirms that family confidence fully mediates the relationship between child functioning and caregiver burden. Therefore, enhancing family confidence should be a priority family-level outcome for ECI services.

#### Informed Consent Statement.

Informed consent was obtained from all subjects involved in the study.
